# Social Media, Thin-Ideal, Body Dissatisfaction and Disordered Eating Attitudes: An Exploratory Analysis

**DOI:** 10.3390/ijerph16214177

**Published:** 2019-10-29

**Authors:** Pilar Aparicio-Martinez, Alberto-Jesus Perea-Moreno, María Pilar Martinez-Jimenez, María Dolores Redel-Macías, Claudia Pagliari, Manuel Vaquero-Abellan

**Affiliations:** 1 Departamento de Enfermería, Universidad de Córdoba, Campus de Menéndez Pidal, 1470 Córdoba, Spain; 2 Usher Institute of Population Health Sciences and Informatics, University of Edinburgh, Edinburgh EH8 9YL, UK; 3 Grupo Investigación epidemiológica en Atención primaria (GC-12) del Instituto Maimónides de Investigación Biomédica de Córdoba (IMIBIC), Hospital Universitario Reina Sofía, 14071 Córdoba, Spain; mvaquero@uco.es; 4 Departamento de Física Aplicada, Universidad de Córdoba, ceiA3, Campus de Rabanales, 14071 Córdoba, Spain; aperea@uco.es (A.-J.P.-M.); fa1majip@uco.es (M.P.M.-J.); 5 Departamento Ingeniería Rural, Ed Leonardo da Vinci, Campus de Rabanales, Universidad de Córdoba, Campus de Excelencia Internacional Agroalimentario, ceiA3, 1470 Cordoba, Spain; mdredel@uco.es; 6 eHealth Research Group, Usher Institute of Population Health Sciences and Informatics, University of Edinburgh, Edinburgh EH8 9YL, UK; claudia.pagliari@ed.ac.uk

**Keywords:** social media, disordered eating behaviours, body image, female, university students

## Abstract

Disordered eating attitudes are rapidly increasing, especially among young women in their twenties. These disordered behaviours result from the interaction of several factors, including beauty ideals. A significant factor is social media, by which the unrealistic beauty ideals are popularized and may lead to these behaviours. The objectives of this study were, first, to determine the relationship between disordered eating behaviours among female university students and sociocultural factors, such as the use of social network sites, beauty ideals, body satisfaction, body image and the body image desired to achieve and, second, to determine whether there is a sensitive relationship between disordered eating attitudes, addiction to social networks, and testosterone levels as a biological factor. The data (*N* = 168) was obtained using validated surveys (EAT-26, BSQ, CIPE-a, SNSA) and indirect measures of prenatal testosterone. The data was analysed using chi-square, Student’s t-test, correlation tests and logistic regression tests. The results showed that disordered eating attitudes were linked to self-esteem (*p* < 0.001), body image (*p* < 0.001), body desired to achieve (*p* < 0.001), the use of social media (*p* < 0.001) and prenatal testosterone (*p* < 0.01). The findings presented in this study suggest a relationship between body image, body concerns, body dissatisfaction, and disordered eating attitudes among college women.

## 1. Introduction

Mental health problems have increased, especially among young people, over the last decade [1]. The most common mental problems are behavioural, emotional, and hyperkinetic disorders. Among these illnesses, disordered eating behaviours are rapidly increasing in a short time, especially among young women [2,3]. These disordered attitudes are defined as afflictions in which people suffer severe disruption in their eating behaviours, thoughts and emotions. The people who suffer from these complaints are usually preoccupied with food and weight. In this sense, disordered eating is used to describe a range of irregular eating behaviours that may or may not warrant a diagnosis of a specific disordered eating attitude [4].

These disorders usually occur in women in their twenties or during adolescence [3]. People who suffer these disorders usually present altered attitudes, behaviours, weight perception and physical appearance [5]. Moreover, disordered eating behaviours or attitudes are defined as unhealthy or maladaptive eating behaviours, such as restricting or binging and/or purging [6]. These behaviours are not categorized as an eating disorder, though they are considered a phase of diagnosed eating disorders [7].

The concern from health care systems is based on the fact that these severe mental disorders usually puts in danger the well-being and health of the people who suffer them [5]. One-third of the women in the world have suffered from these mental problems at some point in their life [6]. If they are inadequately treated, they may develop severe clinical disorders [8]. Moreover, around 1% of the people with these disordered eating attitudes struggle with unhealthy and emotional problems through all their lives [6].

Out of the population with disordered eating attitudes, 16% of them present overeating, 20% purged by vomiting, and 61% food restraining [9]. These frequencies changed as people aged, with food restriction being more common in older women and vomiting during adolescence [10]. Moreover, recent data have discussed the increase of how the minimum age of the people with disorders is around 12 years of age and decreasing. Meanwhile, the prevalence of disordered eating attitudes appears to increase as young adults or adolescents grow older [10].

Although these diseases have a crucial psychobiological component, social and cultural factors have a significant influence. Among these factors, advertising has been described as an internalizing or normalizing means to spread unrealistic beauty ideals. Therefore, a higher incidence of these diseases is presented in advanced and modern societies and people with the best living conditions, mostly caused by the popularization of thin and muscular ideals [11,12,13].

Several biological factors have been linked to disordered eating attitudes, with up to 50% of disordered eating being described as familiarly transmitted [5,14]. Researchers have also suggested that neurotransmitters in the brain are involved in disordered eating attitudes and, therefore, eating disorders [15,16]. Additionally, the hormones have been linked as factors to puberty, body perception and body concerns [17,18]. Testosterone is included among those hormones highly studied, with blood samples providing a more precise method of examination. Nevertheless, different researchers pointed out the possibility of using indirect markers to avoid taking biological samples and creating risks for the participants. In this sense, most studies have linked testosterone and estrogenic levels via the 2D:4D digital ratio as an indirect indicator [19], which heavily dictates attractiveness [17]. This ratio, which is based on the difference in length of the phalanges of the hands (2D:4D ratio) having a lower ratio as an indicator of the existence of a higher level of testosterone, is used for the determination of intrauterine testosterone levels during gestation [20]. This ratio has reflected the relationship with self-perception, body image, body dissatisfaction, and disordered eating behaviours [20,21]. Based on these studies, the hormone levels, and the indirect marker, might appear to have essential roles in disordered eating attitudes [22]. Nevertheless, other authors have described how biological or genetic factors are essential, but may not determine, these disordered eating attitudes [23].

Other factors, such as ethical or familiar factors, contribute to the development of this disordered eating behaviours [24]. In this sense, previous studies have established that the probability of developing a disordered eating attitude or a diagnosis of eating disorders is higher if the mother had a disordered eating or self-esteem problems [25,26]. Moreover, ethnicity has been linked to the perception of beauty ideals, self-esteem and body perception [27,28].

Another critical factor is the media by which beauty ideals have been promoted. The media plays a vital role in formulating what is attractive in society, increasing the thin beauty ideal among females being unattainable [29,30]. These ideals confirmed the way young people perceived themselves and, therefore, how they value themselves [10,31]. This contradiction between what society portrays as a role model and the real body that many young women have has resulted in body concerns. Body concerns usually maintain over time and increase body dissatisfaction. This body dissatisfaction emerges because of the distortion on the body image, its perception and, therefore, body concern [32,33]. This dissatisfaction also plays an essential role in disordered eating attitudes since it provokes emotional and psychological distress [34].

In this sense, the theory of social comparison and numerous studies have studied the relationship between body dissatisfaction and disordered eating attitudes to better understand the causes of these illnesses. These previous works showed that real comparisons with other people leads to a distortion of body image and may favour disorderly feeding [11,29,35]. Additionally, Fredrickson and Roberts (1997) suggested that sexualization and self-objectification promoted via media should be considered as a risk factor for disordered eating attitudes [36,37,38]. Based on previous and recent studies it seems that the role of the media in disordered eating attitudes is noteworthy [1,11,39].

This paper presents a research study in which these objectives have been pursued: first, to determine the relationship between disordered eating attitudes in female university students and sociocultural factors, such as the use of social network sites, beauty ideals, body satisfaction, the body image and the body image desired to achieve. Second, to determine whether there is a sensitive relationship between disordered eating attitudes, addiction to social networks, and other biological factors, such as testosterone levels.

## 2. Background

College-aged women may be at particular risk for body dissatisfaction and disordered eating practices due to the unhealthy weight gain that often occurs during this life stage [3,31]. The promotion of beauty ideals in the media disseminates disordered eating [40,41], drive for thinness and body dissatisfaction among female college students [42]. Furthermore, the growth of social networking sites (SNS), such as Facebook or Instagram, has also increased the exposure to thin and fit ideals [2,43,44]. The social media are more used than any other media as a mean of communication. These internet-based sites pulled the users to create personal profiles and share, view, comment and ‘like’ peer-generated content [20].

Importantly, young people, almost 90% of them (ages 18–29), reported being active users and being continuously exposed to different content and images in this medium [14,45]. Among the most active users of these media stands out the influencers. These new media role models have a significant impact in the last tendencies, the news and the trends that young people are following [46]. In this sense, researchers have also pointed out how social media and influencers may have the key to decrease body dissatisfaction and body concerns. Nevertheless, substantial studies have shown that economic interests are linked with the promotion of dieting in social media, or even surgery [47].

The last publications concluded that the most dangerous social media was Instagram, followed by Facebook and Twitter. These conclusions were based on the instant satisfaction of reviewing and having peer views in the images posted by the users [48]. Especially on Instagram, the message is accommodated according to the image uploaded [47].

These studies concluded that the influence of the advertising and the promotion of the thin and muscular ideals might more be connected with the perception that young people has regarding body, dieting and social media [49]. Additionally, the objectification suggests that the media’s sexual objectification of women modifies their body appearance. Due to this, it could be concluded that self-perception slowly shapes attractiveness resulting in a modification in the body-image, body dissatisfaction and disordered eating attitude. That being said, the proposed hypotheses are as follows:

**Hypothesis 1** **(H1).** *Among young women, self-image will be linked to body dissatisfaction, the thin-ideal and the desire to change one’s body shape.*

**Hypothesis 2** **(H2).** *The level of body dissatisfaction among female college students will be high and be linked to self-esteem.*

**Hypothesis 3** **(H3).** *The young women’s eating behaviours will be linked to the degree of body dissatisfaction and the frequency of using social media.*

**Hypothesis 4** **(H4).** *The young women’s body image and body description will be slightly connected to prenatal testosterone levels.*

## 3. Methodology

### 3.1. Design and Sample

In the first phase, a cross-sectional study was carried out focused on female college students, aged from 18 to 25 years. The sample was recruited to participate in an in-person survey from April to May 2018 from the University of Cordoba. The selection of the sample was based on non-probability convenience sampling. This method of sampling was selected based on the accessibility of the students and previous scheduling with the professors.

The final sample was constituted by 168 subjects, from biological, education, informatics and nursing degrees who agreed to participate in the study voluntarily. The initial sample was 224, though the final sample was 168 after applying the exclusion terms. The mean age of the sample was 20 ± 0.76.

### 3.2. Measures

All the surveys used in the study are validated in different languages, including Spanish. Moreover, these surveys are used globally among health professionals and researchers in the health field [50].

The demographic and anthropometric data were not included in this study since the objective focused on the socio-cultural and individual factors. In this sense, the perception of young people was focused on social media, self-appearance, specific social network sites and distorted eating behaviours.

The EAT-26 with the reduced version of 26 items, was used to assess the frequency of disordered eating attitudes [51,52]. This test measures the low, medium and high risk of having a disordered eating attitude. Moreover, three different disordered eating behaviours can be reflected depending on the answers to each item. In this sense, these three subscales are dieting (focused on questions 1, 6, 7, 10, 11, 12, 14, 16, 17, 22, 23, 24, 26), bulimia and food preoccupation (focused on questions 3, 4, 9, 18, 21, 25) and food oral control (2, 5, 8, 13, 15, 8, 20). Total scores were calculated by taking the sum of the 26 items, based on the value from 0 to 3, where higher scores, over 20 points, indicated higher levels of disordered eating behaviours. This validated survey based on screening disorder eating attitudes when the score is over 20 points [52]. Nevertheless, this survey does not provide a definite diagnosis of eating disorders; therefore, a clinical evaluation is needed. This evaluation can be carried out via individual interviews.

The body satisfaction questionnaire (BSQ) [53], whose Spanish adaptation was completed by Raich [54], was used. The stereotypes perception survey from the University of Granada was also used [55].

The questions referring to body image included illustrations of women’s bodies. These illustrations comprise seven body images that vary from underweight to obese, numbered from 1 to 7. Additionally, a specific section focused on body satisfaction, examining their satisfaction on a scale from 1 to 7, with lower scores relating to higher levels of body dissatisfaction. In this section, one of the questions examined the steps each young person would take to attain a body type that corresponded to the ideal.

The body image concerns were observed by using the BSQ, a self-report instrument evaluating weight and shape preoccupations [54]. Sample items include: “Have you been so worried about your shape that you have felt you ought to diet?”; “Have you noticed the shape of others and felt that your shape compared unfavourably?” The questions were answered on a six-point Likert scale (1 = never, five = always).

The Appearance Evaluation (AE) subscale of the Multidimensional Body-Self Relations Questionnaire-Appearance Scales (MBSRQ) was used to measure self-perception and stereotypes [56]. Participants rate the extent to which they agree with seven statements (e.g., “Most people would consider me good-looking”) on a five-point scale (1 = disagree, 5 = agree) with lower scores indicating lower self-perception and stereotypes.

Finally, self-esteem was evaluated by the Rosenberg survey (CIPE-a) composed of ten questions, which provided us with high, medium or low levels of self-esteem. The questions were given a scale on a four-point scale (1 = disagree, 4 = agree), with lower scores indicating lower self-esteem [57].

On the other hand, the survey that focused on social networks had preliminary yes/no items about having social network accounts on Twitter, Facebook, Instagram, YouTube or Snapchat. Participants indicated how often they access/check their respective accounts daily on a five-point scale: hardly ever, sometimes, usually, all most all the time and always. Additionally, the participants’ daily use (hours per day in social networks and highly visual social media, i.e., Instagram, Snapchat), number of accounts and importance given to these was rated on a 1 (strongly disagree) to 5 (strongly agree) scale.

Meanwhile, addiction to social networks was evaluated by a validated survey called the Social Networks Addiction Questionnaire (SNSA) [50]. The survey is based on the DSM-IV-TR [27], a diagnostic instrument that does not recognize psychological addictions as disorders but as a prior stage that can lead to addiction. The survey is formed by 24 items applying a five-point rating system (from 0 to 4), taking into account the frequency from “never” to “always” [56].

The study has focused on the indirect determination of intrauterine testosterone levels during the gestation, determined experimentally from the difference in length of the phalanges of the hands (2D:4D ratio). This measure was selected to determine the possible relation with sociocultural factors indirectly. The selection of this method was based on reducing the risks, vulnerability and protecting biological or genetic material from the participants. When the ratio is higher, i.e., the difference between the second and fourth finger, lower levels of testosterone are implied [21]. 2D:4D is an indicator of testosterone and oestrogen levels [58], which heavily dictate attractiveness [17]. Therefore, this digit ratio may be related to self-perception, body image, body dissatisfaction and disordered eating attitudes.

### 3.3. Instruments

The instruments used to obtain the image of the hands were a Canon Camera EOS700D (produced by Canon Inc., which is a Japanese company founded in Ota, Tokyo) and a Manfrotto Compact Advance tripod (produced by Manfrotto, which is an Italian company founded in, produced and distributed form the USA). Additionally, free access software GeoGebra (https://www.geogebra.org), which is a free access software founded in Austria and later updated and mass produced in USA, was used to analyse the indirect marker of testosterone levels (2D:4D ratio).

### 3.4. Procedure

Participants approved a participant information statement, consent form and questionnaires, followed by the approval of the Research Ethics Committee of Public Health System in Cordoba (Ethical Approval number 273, reference 3773).

The participants were undergraduate students with health, education, life and engineering studies. The recruitment took place in different classrooms of the University, the objective of the study, ethical indications, risks for the participants and voluntary participation in the study being previously explained. During the recruitment a teacher and a researcher were present in the classroom the entire time.

**Figure 1:**
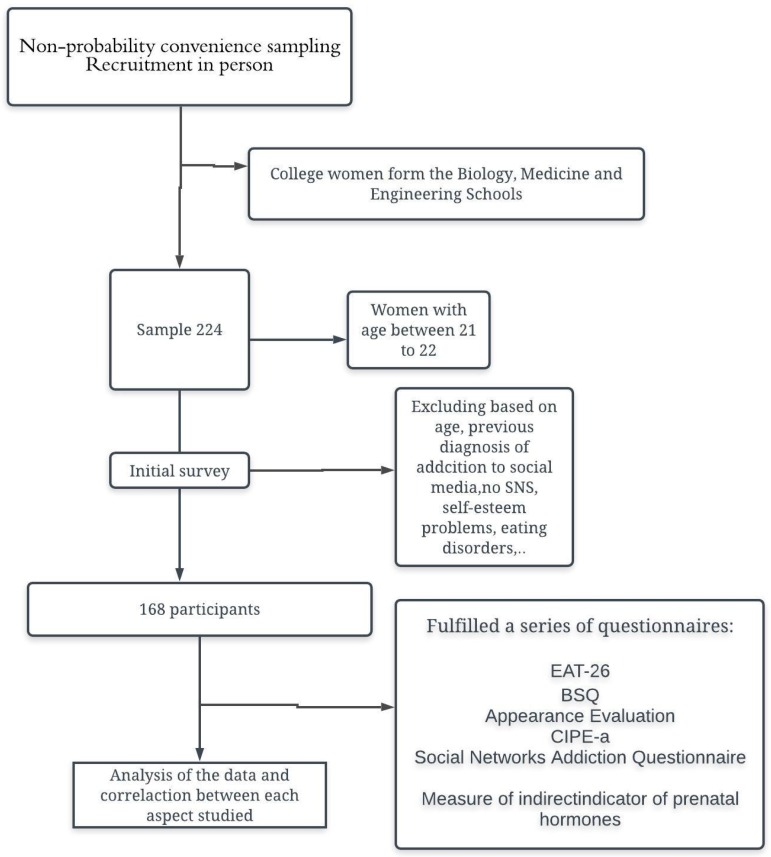
Flow chart of the recruitment and selection of the sample.

The inclusion of the participants was based on an initial survey, which was provided previously in the same classroom. In this survey, the students were asked about the previous diagnosis of conduct or emotional disorders, addiction to technologies, abuse of substances and having a social network account. Those students that had a previous diagnosis of conduct, emotional disorders, or addiction were eliminated from the sample and were not given the survey of the study. Those students that did not have an account on any social network were also excluded from the study (Figure 1).

### 3.5. Statistical Analysis

Mean and standard deviation (SD) were calculated for the quantitative variables and frequencies in the case of qualitative variables. Firstly, we studied the normalization of the data using the Kolmogorov-Smirnov test (*p* < 0.05). Moreover, Cronbach’s alpha test was used for determining the consistency among the scales and subscales and, especially, the SNS test showed acceptable value (0.77) and the EAT-26 (0.83) was excellent. In order to assess the first objective, the χ^2^ test was used for the qualitative variables, such as gender and body image, and the Student’s *t*-test was applied to compare quantitative variables, such as the EAT-26 score and age. Additionally, correlational analyses were used to examine relations between all variables.

Moreover, the second set of analyses examined the impact of the relationship between disordered eating attitudes and the rest the factors measured. For this purpose, the crude and adjusted odds ratio (OR) values were calculated for the logistic regression. In the end, the ROC (receiver operating characteristic) curves and the validity indices were used for the diagnostic accuracy of disordered eating attitudes having body dissatisfaction and social networks addiction.

## 4. Results

### First Phase

The initial analysis of the data showed that women (*N* = 168) had a range of age between 21 and 22, 96.7% of them being Caucasian ethnicity. Moreover, the body image that they had was in range between 3 and 4, which may imply a normal weight. The perception that they had of themselves was fatter (3.56 ± 1.2) when compared to the desired body image (2.99 ± 0.83) (Table 1). Additionally, the most common description of body satisfaction showed low and medium-high levels of body satisfaction (48.7%). In this sense, the difference among the group with lower and higher levels of body satisfaction was related to the body image given by the women (χ^2^ = 113.64, *p* < 0.001).

Moreover, the results from the data showed that almost 93% of the women desired to change at least three zones of their body using at least two different methods (1.98 ± 0.82). The methods most used were physical activity (92%), diet (48%), surgery (24%) and beauty or alimentary products (23%). Among the zones to be modified by a surgical procedure 68% of the women indicated breast implants.

**Figure 2:**
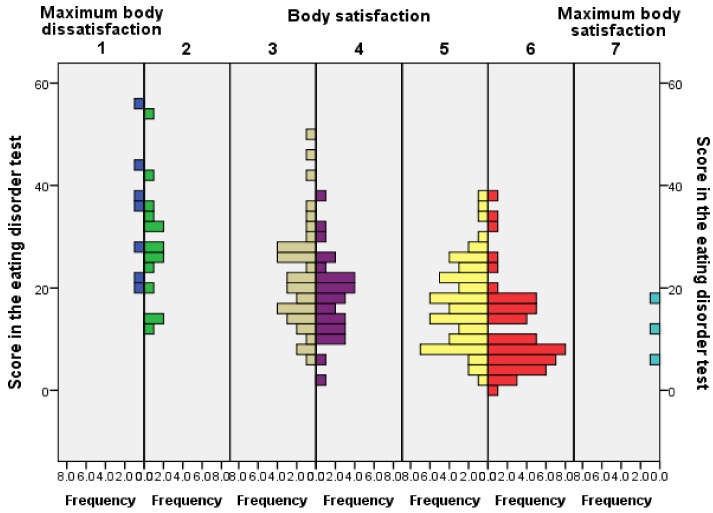
Results from the EAT-26 related to body satisfaction.

The analysis of the results from the EAT-26 test showed that most of the women had a medium probability of having disordered eating attitudes (18.34 ± 10.7). Figure 2 reflects the frequency of the scores from the EAT-26 related to body satisfaction.

The figure displays a higher frequency of scores over 20 points in disordered eating behaviours in the lower points of the body satisfaction scale. This figure implies that there were more values over 20 points when women suffered higher levels of body dissatisfaction. Additionally, the analysis between the score in the disordered eating behaviour test and level of body satisfaction showed significant differences among individuals with low and high levels of body satisfaction and scores over 20 points in the EAT-26 (χ^2^ = 375.34, *p* < 0.001). Moreover, a more in-depth analysis of the data, based on women with more than 20 points in the EAT-26, 48 out of 168 women showed that 40.81% had food oral control, 38.77% presented bulimia and food preoccupation and 20.5% dieting.

Further study of the data was carried out in order to address the possible correlations between the body image that women perceived of themselves and the other variables analysed. In Table 2, the correlations between the body image and the different variables have shown significant value with numerous factors, including disordered eating attitudes, self-esteem, desired body image or number of methods. These correlations were positive for a fatter body image in higher scores in the EAT-26 and more methods used to modify the body image and the current body image. Moreover, negative correlations were found for a curvier description that the women gave about their body and higher desires for a thinner body image, higher body dissatisfaction and lower levels of self-esteem.

Another variable that determines a “fatter” body image is the level of prenatal testosterone, measured by the 2D:4D ratio. This result displayed a positive relationship implying that a higher 2D:4D ratio, lower levels of intrauterine testosterone, may lead to a fatter body image.

On the other hand, Table 3 exposed the analysis of correlations between the score obtained in EAT-26 for disordered eating attitudes and the other factors analysed. This test displayed a negative correlation between having a higher score in the test and having lower levels of body satisfaction, self-esteem, the desired of having a thinner body image and worse perception of their own body.

Moreover, the positive correlations were obtained for numerous factors studied. The most highlighting positive correlations were reflected for a higher score in the SNS addiction test, a fatter body image and a higher difference in the 2D:4D ratio. These results implied that a higher 2D:4D ratio or fatter body image may lead to a higher score in the EAT-26.

The logistic regression model was used to define a disordered eating behaviour related to having lower levels of body satisfaction, the desired to achieve a thinner body image, lower levels of self-esteem, higher score in the SNS addiction test, higher duration of connection to this media and higher difference between the second and fourth finger (Table 4).

From the analysis based on levels of self-esteem and social networks, the results showed that most women have high levels of self-esteem (31.1 ± 4.7) and low levels of addictive behaviour to social network sites (14.69 ± 10.37). Furthermore, the results of the social network sites presented a high dispersion of the results. In this sense, the confidence intervals (95%) were focused on medium levels regarding addictive behaviour to SNS (13.11–16.26).

Based on this, the correlations for the score in the SNS addition test were studied. The results indicated positive significance for the number of methods used to change their body image (<0.001), higher desired of a thinner body (*p* < 0.001), lower levels of self-esteem (*p* < 0.001), greater number of social media accounts (*p* < 0.001), longer duration of the connections (*p* < 0.001) and the importance given to the social networks (*p* < 0.001). Nevertheless, the difference between the second and fourth phalange (2D:4D ratio) showed no significance with scores in the social network addiction test.

**Figure 3:**
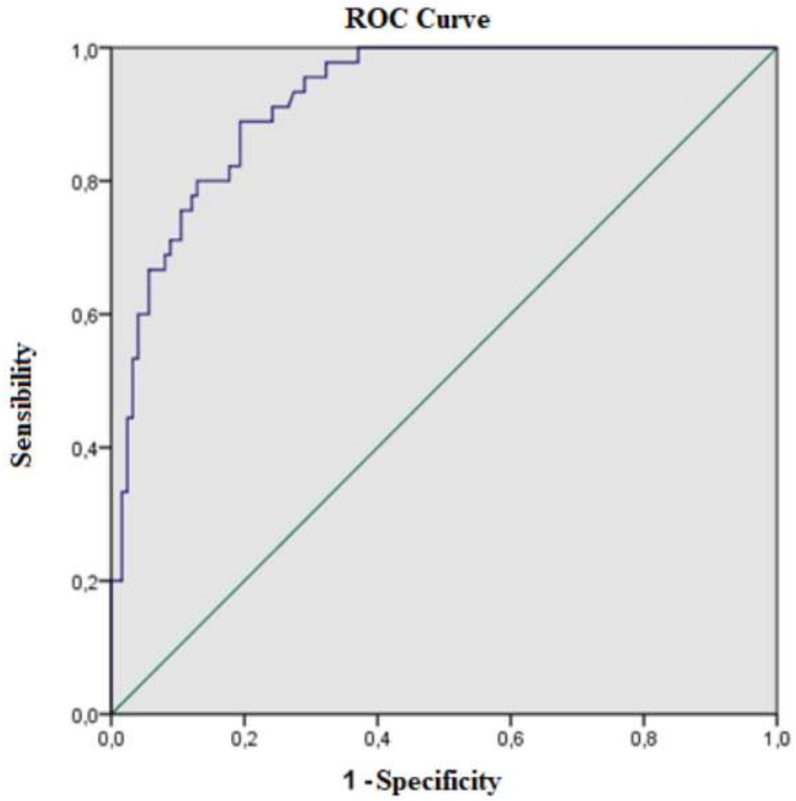
ROC curve from the logistic model for disordered eating.

Finally, based on the results from the logistic regression, a probabilistic model was obtained. This model could diagnose 42.9% of the population with disordered eating attitudes (R^2^ Cox and Snell 0.429) by knowing if the person had scored high in the SNS addiction test, body image, body dissatisfaction and high desire of having a thinner body. The specificity (90.3), sensibility (68.9) and valid index (84.6) results were optimal. Finally, the curve of the model was analysed (Figure 3) obtaining an acceptable probabilistic high risk of a disordered eating attitudes (area = 0.94, *p* < 0.001, CI 0.88–0.97).

## 5. Discussion

This study has reflected how different factors, such as the level of self-esteem (Table 1), might play a significant role in disordered eating behaviours. Among these factors the body image that women perceived over themselves stood out as a significant element. In this sense, according to previous researchers, body image is multidimensional, being made of perceptual, behavioural and cognitive-affective domains created by the individual [46]. This perception is dependent on a variety of elements, including social media and beauty ideals. In the case of social media, the results from this study showed a relationship between the body image, body ideals and the use of social media (Table 2 and Table 3). Furthermore, previous publications explained that the desire to achieve the beauty ideal emerges as the internalization of the portrayed image exposed by the media [59,60]. Homan (2010) discussed how, among female college students, two principal beauty ideals coexist: the athletic-ideal and thin-ideal [61]. The internalization of the athletic-ideal predicts compulsive exercise [61,62,63]. Meanwhile, the thin-ideal internalization predicts food restriction and body dissatisfaction, both leading to disordered eating attitudes and possible origins for eating disorders [64,65,66]. These results confirm the association obtained between the desire of having a thinner body image and the use of the media since this media is the primary source to promote such ideals (Table 3).

The issue resides on the fact that the thin-ideal produces a worse body image with a tendency toward frustration based on a fatter body image than desired. This concern among young women results in making different choices to obtain the desired image, such as surgery [67,68]. In this sense, the results from this paper also showed a high frequency of women determined to undergo plastic surgery to improve their image, being focused on breast surgery.

Notwithstanding, internalization of the fit-ideal has been studied as a predictor of the use of social media content related to health and fitness [69,70]. In this case, the fit ideal or athletic ideal may become a replacement for the other ideals, leading to healthier behaviour [71].

The results (Table 2) have established that body dissatisfaction might be a potential agent in body image and desire to change this body image. These publications also accord with our earlier observations, which showed that levels of body dissatisfaction were associated with the desire of changing the body image in order to achieve a thinner body, especially using dieting [72]. Based on this, the results appear to match with previous works about how body dissatisfaction and body concerns in young women and teenagers may be related to disordered eating attitudes [27,73].

Another significant outcome was the link between body concerns, body dissatisfaction and levels of self-esteem (Table 2). These data are in accord with recent investigations which connected body dissatisfaction and self-esteem to mental illness and the role of emotional distress in behavioural disorders [48].

Another study found that body dissatisfaction and disordered eating attitudes could be related to a high level of intrauterine testosterone, measured by the 2D:4D ratio. The prenatal masculinization has been established as a potential intermediate phenotype for the development of these disorders in their offspring [74]. Following these studies, the results obtained in this paper seem to initially match such conclusions (Table 3) [75]. These results are partially consistent with the existing literature relating to dieting, alimentary products, such as supplements, negative affect, body dissatisfaction and the tendency to thinness [71]. Nevertheless, the results obtained regarding the hormonal levels may be related to the environmental conditions during the pregnancy more than the individual level of hormones [76].

The results of the study (Table 4) have shown how social network sites might play an important role in disordered eating attitudes. In the study carried out by Cohen et al. (2018), the influence of the social networks was determined by the content and the selfies that the users upload to them more than by the assiduity of the connections [20]. This is partially contradictory to the present results in which the addiction to SNS and the duration of the connections were linked to weight loss and unhealthy dieting. These results match with previous studies in the sociocultural factors, not included among biological measures [77,78]. Withstanding, it is important to note that the regression model obtained in this study have shown the probable role of factors, such as the degree of body satisfaction, self-esteem, use of SNS and other measures, such as the 2D:4D ratio, related to disordered eating behaviours.

Additionally, SNS addiction, which has been related to other mental disorders [79], has shown correlation with stereotypes, self-esteem, method of change, thinner body image and the desired part of the body to change. In this sense, prior investigations proved the addiction to social media as cause–effect of disordered behaviours [80,81].

The present study raises the possibility that disordered eating attitudes in women might be conditioned by the influence of the ideals of beauty imposed by the social environment and to a lesser extent by the exposure to intrauterine levels of testosterone extracted from the 2D:4D ratio of the phalanges. It is possible, therefore, that disordered eating attitudes are multidimensional disorders produced by the media, hormones, and factors related to body concerns. Although this study has focused on Spanish college students, the results (Table 2 and Figure 2) seem to match with previous works conducted in Caucasian women [82,83]. These studies seem to distant themselves from publications focused on Latina or African American young women or adolescents [84,85]. Nevertheless, it is possible, therefore, that because the study was carried out in Spanish college students, the results might not match university women from other countries.

Nevertheless, as with all research, the current findings need to be considered in light of possible limitations of the study. Therefore, biases and possibly incorrect data may have been included, and causal inferences cannot be drawn. Additionally, as with the majority of the body image literature, the current participants were university students, based on the sample and size of the sample caution is recommended in not generalizing these results to other samples or different samples. Nevertheless, these results seem to provide essential data regarding social media, disordered eating and the perception of the young people about themselves. Another limitation present in this study is the lack of inclusion of further cultural factors, such as the mother–child relationship, and anthropometric data, such as BMI.

All being said, the results from this manuscript and the comparison with previous works suggest how the initial hypothesis has been entirely or partially confirmed, showing how disordered eating behaviours are complex eating attitudes.

## 6. Conclusions

This paper has argued the relationship between body image, body concerns, body dissatisfaction, and disordered eating behaviours present in college women from the south of Spain. This study has identified that women reported moderate levels of body dissatisfaction and body concerns, which were consistently and strongly associated with disordered eating attitudes. In this sense, this work has established high levels of body dissatisfaction, and the link with the desire to achieve a thinner body image. Additionally, the study has shown how body dissatisfaction and desire to achieve the thin-ideal appear to be universal among college women.

Additionally, one of the more significant findings to emerge from this study was that the thin-ideal seems to be widespread in social media. This ideal can promote unhealthy measures, such as dieting, increase body dissatisfaction and disordered eating attitudes. In this sense, the desire to change the body image and taking unhealthy measures was common, given the proliferation of the use of the social network sites where images and content encourage women to aspire to unrealistic and unattainable body ideals. In this sense, the study associated body dissatisfaction, body concerns, and general mental well-being, demonstrating that interventions to improve body perception and satisfaction are essential. Additionally, this research found that higher levels of prenatal testosterone might decrease the probability of having a disordered eating attitude among women. That said, the current study suggests a connection between disordered eating attitudes, negative impacts of exposure to thin-ideal content, addiction to social media and intrauterine testosterone levels.

Concerning practical implications, researchers have asserted that increasing body appreciation may be easier than attempting to decrease body dissatisfaction and for those disordered eating attitudes. Furthermore, the findings regarding the negative impact of exposure to social media related to women’s body satisfaction and body appreciation are notable. Despite the limitations present in this manuscript, the findings may help us to understand body concerns focused on the impact of exposure to social media.

In the end, future investigations should continue exploring differences in the levels of body dissatisfaction and disordered eating, including the differences between various ethnic groups. Given the findings regarding differences between those with higher and lower score in EAT-26, the role of social media may be essential in levels of body dissatisfaction and disordered eating attitudes within specific gender/age groups. Longitudinal research is needed to determine the direction of the association between the frequency of connections to social media and body dissatisfaction/disordered eating behaviours. Researchers may also consider culturally-relevant factors that may differentially influence such behaviours.

## Figures and Tables

**Table 1:** Mean, standard deviation and confidence intervals.

Factors Studied in Women	Mean (SD)	CI 95%
Self-image	3.56 (1.2)	3.38–3.75
Disordered eating	18.34 (10.7)	16.70–19.97
Self-description	3.99 (0.98)	3.84–4.14
Body satisfaction	4.32 (1.48)	4.1–4.54
Desired body image	2.99 (0.83)	2.86–3.12
Method of change	1.98 (0.82)	1.76–2.01
Zone to change	3.37 (1.95)	3.08–3.67
Self-perception	2.76 (0.89)	2.62–2.89
Stereotypes	2.59 (0.75)	2.48–2.71
Self-esteem	31.10 (4.7)	30.3–31.8
SNS addiction	14.69 (10.37)	13.11–16.26
Use of social media	3.13 (0.72)	3.02–3.24
Frequency of connections	3.46 (0.92)	4.32–3.6
Duration of the connections	3.2 (1.17)	3.02–3.38
Importance of social media	2.8 (0.87)	2.67–2.94

**Table 2:** Correlations with body image that women perceived of themselves.

Factors Studied in Women	Correlation	*p*-Value
Disordered eating attitudes	0.29	<0.001
Self-description	0.72	<0.001
Body satisfaction	−0.39	<0.001
Desired image	−0.46	<0.001
Method of change	0.22	<0.01
Self-perception	−0.38	<0.001
Stereotypes	0.38	<0.001
Self-esteem	−0.34	<0.001
2D:4D ratio	0.17	<0.05

**Table 3:** Correlations with having higher scores in the disordered eating attitudes test.

Factors Studied in Women	Correlation	*p*-Value
Body Image	0.32	<0.001
Self-description	0.34	<0.001
Body satisfaction	−0.64	<0.001
Method of change	0.37	<0.01
Self-perception	−0.38	<0.001
Stereotypes	0.57	<0.001
Desired image	−0.19	<0.05
Zone to change	0.35	<0.001
Self-esteem	−0.49	<0.001
Addiction to SNS	0.18	<0.05
2D:4D ratio	0.41	<0.001

**Table 4:** Logistic regression for disordered eating attitudes.

	Non-Adjusted				Adjusted	
Factors Studied in Women	ED (Yes)	ED (No)	OR	CI	OR	CI
Body Dissatisfaction	3.35 (1.48)	4.73 (0.21)	0.49	0.38–0.64	0.54	0.33–0.87
Desired image	2.76 (0.14)	3.19 (0.07)	0.56	0.37–0.83	0.24	0.11–0.52
Stereotypes	3.15 (0.69)	2.35 (0.98)	5.17	2.95–9.06	2.56	1.16–0.65
Self-esteem	2.80 (0.53)	3.26 (0.39)	0.10	0.043–0.24	0.15	0.04–0.63
Addiction to SNS	0.96 (0.11)	0.68 (0.57)	1.71	0.56–0.74	0.48	0.23–1.01
Duration of the connections	3.43 (0.17)	3.03 (0.09)	1.32	1.4–1.76	1.68	1.05–2.69
Testosterone levels (2D:4D ratio)	1.20 (0.79)	0.6 (0.79)	2.49	1.62–3.62	3.13	1.60–6.12

## References

[1] Dowds J. (2010). What do young people think about eating disorders and prevention programmes? Implications for partnerships between health, education and informal youth agencies. JPMH.

[2] Fardouly J., Vartanian L.R. (2015). Negative comparisons about one’s appearance mediate the relationship between Facebook usage and body image concerns. Body Image.

[3] Bailey A.P., Parker A.G., Colautti L.A., Hart L.M., Liu P., Hetrick S.E. (2014). Mapping the evidence for the prevention and treatment of eating disorders in young people. J. Eat. Disord..

[4] Telch C.F., Pratt E.M., Niego S.H. (1997). Obese women with binge eating disorder define the term binge. Int. J. Eat. Disord..

[5] Plateau C.R., Brookes F.A., Pugh M. (2018). Guided recovery: An interpretative phenomenological analysis of service users’ experiences of guided self-help for bulimic and binge eating disorders. Gogn. Behav. Pract..

[6] Reba-Harrelson L., von Holle A., Hamer R.M., Swann R., Reyes M.L., Bulik C.M. (2009). Patterns and prevalence of disordered eating and weight control behaviors in women ages 25–45. Eat. Weight Disord..

[7] Croll J., Neumarksztainer D., Story M., Ireland M. (2002). Prevalence and risk and protective factors related to disordered eating behaviors among adolescents: Relationship to gender and ethnicity. J. Adolesc. Health.

[8] Baranowksi M.J., Jorga J., Djordjevic I., Marinkovic J., Hetherington M.M. (2003). Evaluation of adolescent body satisfaction and associated eating disorder pathology in two communities. Eur. Eat. Disord. Rev..

[9] Harris J.K., Duncan A., Men V., Shevick N., Krauss M.J., Cavazos-Rehg P.A. (2018). Messengers and messages for tweets that used #thinspo and #fitspo hashtags in 2016. Prev. Chronic Dis..

[10] Garousi S., Garrusi B., Baneshi M.R., Sharifi Z. (2016). Weight management behaviors in a sample of Iranian adolescent girls. Eat. Weight Disord..

[11] Kraak V.I., Story M. (2015). Influence of food companies’ brand mascots and entertainment companies’ cartoon media characters on children’s diet and health: A systematic review and research needs. Obes. Rev..

[12] Otero M.C., Fernández M.L., Castro Y.R. (2004). Influencia de la imagen corporal y la autoestima en la experiencia sexual de estudiantes universitarias sin trastornos alimentarios. Int. J. Clin. Health Psychol..

[13] Calado M., Lameiras M., Sepulveda A.R., Rodríguez Y., Carrera M.V. (2010). The mass media exposure and disordered eating behaviours in Spanish secondary students. Eur. Eat. Disord. Rev..

[14] Rodgers R.F., Faure K., Chabrol H. (2009). Gender differences in parental influences on adolescent body dissatisfaction and disordered eating. Sex. Roles.

[15] Walsh B.T., Devlin M.J. (1998). Eating disorders: Progress and problems. Science.

[16] Muris P., Meesters C., van de Blom W., Mayer B. (2005). Biological, psychological, and sociocultural correlates of body change strategies and eating problems in adolescent boys and girls. Eat. Behav..

[17] Wade T.J., Shanley A., Imm M. (2004). Second to fourth digit ratios and individual differences in women’s self-perceived attractiveness, self-esteem, and body-esteem. Personal. Individ. Differ..

[18] Guo S.-W., Reed D.R. (2012). The genetics of phenylthiocarbamide perception. Ann. Hum. Biol..

[19] Manning J., Barley L., Walton J., Lewis-Jones D., Trivers R., Singh D., Thornhill R., Rohde P., Bereczkei T., Henzi P. (2000). The 2nd: 4th digit ratio, sexual dimorphism, population differences, and reproductive success: Evidence for sexually antagonistic genes?. Evol. Hum. Behav..

[20] Eichler A., Heinrich H., Moll G.H., Beckmann M.W., Goecke T.W., Fasching P.A., Muschler M.-R., Bouna-Pyrrou P., Lenz B., Kornhuber J. (2018). Digit ratio (2D:4D) and behavioral symptoms in primary-school aged boys. Early Hum. Dev..

[21] Barut C., Tan U., Dogan A. (2008). Association of height and weight with second to fourth digit ratio (2D:4D) and sex differences. Percept. Mot. Skills.

[22] Jeevanandam S., Muthu P.K. (2016). 2D:4D ratio and its implications in medicine. J. Clin. Diagn. Res..

[23] Breithaupt L., Rallis B., Mehlenbeck R., Kleiman E. (2016). Rumination and self-control interact to predict bulimic symptomatology in college students. Eat. Behav..

[24] Wood N.A.R., Petrie T.A. (2010). Body dissatisfaction, ethnic identity, and disordered eating among African American women. J. Couns. Psychol..

[25] Loeb K.L., Hirsch A.M., Greif R., Hildebrandt T.B. (2009). Family-based treatment of a 17-year-old twin presenting with emerging anorexia nervosa: A case study using the “Maudsley method”. J. Clin. Child Adolesc. Psychol..

[26] Bourke-Taylor H.M., Jane F., Peat J. (2019). Healthy mothers healthy families workshop intervention: A preliminary investigation of healthy lifestyle changes for mothers of a child with a disability. J. Autism Dev. Disord..

[27] Schaefer L.M., Burke N.L., Calogero R.M., Menzel J.E., Krawczyk R., Thompson J.K. (2018). Self-objectification, body shame, and disordered eating: Testing a core mediational model of objectification theory among White, Black, and Hispanic women. Body Image.

[28] Howard L.M., Heron K.E., MacIntyre R.I., Myers T.A., Everhart R.S. (2017). Is use of social networking sites associated with young women’s body dissatisfaction and disordered eating? A look at Black–White racial differences. Body Image.

[29] Festinger L. (1954). A theory of social comparison processes. Hum. Relat..

[30] Powell E., Wang-Hall J., Bannister J.A., Colera E., Lopez F.G. (2018). Attachment security and social comparisons as predictors of Pinterest users’ body image concerns. Comput. Hum. Behav..

[31] Neumark-Sztainer D., Wall M., Larson N.I., Eisenberg M.E., Loth K. (2011). Dieting and disordered eating behaviors from adolescence to young adulthood: Findings from a 10-year longitudinal study. J. Am. Diet. Assoc..

[32] Caradas A.A., Lambert E.V., Charlton K.E. (2001). An ethnic comparison of eating attitudes and associated body image concerns in adolescent South African schoolgirls. J. Hum. Nutr. Diet..

[33] Cohen R., Newton-John T., Slater A. (2017). The relationship between Facebook and Instagram appearance-focused activities and body image concerns in young women. Body Image.

[34] Hoare E., Marx W., Firth J., McLeod S., Jacka F., Chrousos G.P., Manios Y., Moschonis G. (2019). Lifestyle behavioural risk factors and emotional functioning among schoolchildren: The Healthy Growth Study. Eur. Psychiatry.

[35] Perez M., Ohrt T.K., Bruening A.B. (2016). The effects of different recruitment and incentive strategies for body acceptance programs on college women. Eat. Disord..

[36] Moradi B., Huang Y.-P. (2008). Objectification theory and psychology of women: A decade of advances and future directions. Psychol. Women Q..

[37] McKinley N.M., Hyde J.S. (1996). The objectified body conciousness scale development and validation. Psychol. Women Q..

[38] Fredrickson B.L., Roberts T.-A. (1997). Objectification theory: Toward understanding women’s lived experiences and mental health risks. Psychol. Women Q..

[39] Groesz L.M., Levine M.P., Murnen S.K. (2002). The effect of experimental presentation of thin media images on body satisfaction: A meta-analytic review. Int. J. Eat. Disord..

[40] Wyssen A., Coelho J.S., Wilhelm P., Zimmermann G., Munsch S. (2016). Thought-shape fusion in young healthy females appears after vivid imagination of thin ideals. J. Behav. Ther. Exp. Psychiatry.

[41] Brooks K.R., Mond J.M., Stevenson R.J., Stephen I.D. (2016). Body image distortion and exposure to extreme body types: Contingent adaptation and cross adaptation for self and other. Front. Neurosci..

[42] Juarascio A.S., Shoaib A., Timko C.A. (2010). Pro-eating disorder communities on social networking sites: A content analysis. Eat. Disord..

[43] Cohen R., Newton-John T., Slater A. (2018). ‘Selfie’-objectification: The role of selfies in self-objectification and disordered eating in young women. Comput. Hum. Behav..

[44] Hummel A.C., Smith A.R. (2015). Ask and you shall receive: Desire and receipt of feedback via Facebook predicts disordered eating concerns: Facebook disordered eating. Int. J. Eat. Disord..

[45] van den Eijnden R.J.J.M., Lemmens J.S., Valkenburg P.M. (2016). The social media disorder scale. Comput. Hum. Behav..

[46] Quick V.M., Byrd-Bredbenner C. (2014). Disordered eating, socio-cultural media influencers, body image, and psychological factors among a racially/ethnically diverse population of college women. Eat. Behav..

[47] (2019). Instagram clamps down on diet and cosmetic surgery posts. BBC News Technology.

[48] Chae J. (2017). Virtual makeover: Selfie-taking and social media use increase selfie-editing frequency through social comparison. Comput. Hum. Behav..

[49] O’Donnell N.H., Willoughby J.F. (2017). Photo-sharing social media for eHealth: Analysing perceived message effectiveness of sexual health information on Instagram. J. Vis. Commun. Med..

[50] Pichot P., Aliño J.J.L., Miyar M.V. (2001). Manual Diagnóstico y Estadístico de los Trastornos Mentales: DSM-IV.

[51] Garfinkel P.E., Newman A. (2001). The eating attitudes test: Twenty-five years later. Eat. Weight Disord..

[52] Cooper P.J., Taylor M.J., Cooper Z., Fairbum C.G. (1987). The development and validation of the body shape questionnaire. Int. J. Eat. Disord..

[53] Cash T.F. (2004). Body image: Past, present, and future. Body Image.

[54] Raich R.M., Mora M., Soler A., Avila C., Clos I., Zapater L. (1996). Adaptación de un instrumento de evaluación de la insatisfacción corporal. Clin. Salud.

[55] Godoy D. Imagen Corporal. https://test.ugr.es/limesurvey/index.php?sid=22944andlang=es.

[56] Mayaute M.E., Blas E.S. (2014). Construcción Y validación del cuestionario de adicción a redes sociales (ars). Liberabit Rev. Peru. Psicol..

[57] Petersen W. (1965). Society and the adolescent self-image. Morris Rosenberg. Science.

[58] Romero-Martínez A., Moya-Albiol L. (2014). Prenatal testosterone of progenitors could be involved in the etiology of both anorexia nervosa and autism spectrum disorders of their offspring. Am. J. Hum. Biol..

[59] Carrotte E.R., Prichard I., Lim M.S.C. (2017). “Fitspiration” on social media: A content analysis of gendered images. J. Med. Internet Res..

[60] de Vries D.A., Vossen H.G.M. (2019). Social media and body dissatisfaction: investigating the attenuating role of positive parent–adolescent relationships. J. Youth Adolesc..

[61] Homan K. (2010). Athletic-ideal and thin-ideal internalization as prospective predictors of body dissatisfaction, dieting, and compulsive exercise. Body Image.

[62] Kantanista A., Glapa A., Banio A., Firek W., Ingarden A., Malchrowicz-Mośko E., Markiewicz P., Płoszaj K., Ingarden M., Maćkowiak Z. (2018). Body image of highly trained female athletes engaged in different types of sport. Biomed. Res. Int..

[63] de Bruin A.P., Oudejans R.R.D. (2018). Athletes’ body talk: The role of contextual body image in eating disorders as seen through the eyes of elite women athletes. J. Clin. Sport Psychol..

[64] Hawkins N., Richards P.S., Granley H.M., Stein D.M. (2004). The impact of exposure to the thin-ideal media image on women. Eat. Disord..

[65] Harrison K. (2000). The body electric: Thin-ideal media and eating disorders in adolescents. J. Commun..

[66] Alberga A.S. (2018). Fitspiration and thinspiration: A comparison across three social networking sites. J. Eat. Disord..

[67] Larson K., Gosain A. (2012). Cosmetic surgery in the adolescent patient. Plast. Reconstr. Surg..

[68] Ching B.H.-H., Xu J.T. (2019). Understanding cosmetic surgery consideration in Chinese adolescent girls: Contributions of materialism and sexual objectification. Body Image.

[69] Holland G., Tiggemann M. (2016). A systematic review of the impact of the use of social networking sites on body image and disordered eating outcomes. Int. J. Eat. Disord..

[70] Slater A., Varsani N., Diedrichs P.C. (2017). #fitspo or #loveyourself? The impact of fitspiration and self-compassion Instagram images on women’s body image, self-compassion, and mood. Body Image.

[71] Uhlmann L.R., Donovan C.L., Zimmer-Gembeck M.J., Bell H.S., Ramme R.A. (2018). The fit beauty ideal: A healthy alternative to thinness or a wolf in sheep’s clothing?. Body Image.

[72] Barker E.T., Bornstein M.H. (2010). Global self-esteem, appearance satisfaction, and self-reported dieting in early adolescence. J. Early Adolesc..

[73] Griffiths S., Murray S.B., Krug I., McLean S.A. (2018). The contribution of social media to body dissatisfaction, eating disorder symptoms, and anabolic steroid use among sexual minority men. Cyberpsychol. Behav. Soc. Netw..

[74] Quinton S.J., Smith A.R., Joiner T. (2011). The 2nd to 4th digit ratio (2D:4D) and eating disorder diagnosis in women. Personal. Individ. Differ..

[75] Uban K.A., Herting M.M., Wozniak J.R., Sowell E.R. (2017). Sex differences in associations between white matter microstructure and gonadal hormones in children and adolescents with prenatal alcohol exposure. Psychoneuroendocrino.

[76] Seo D., Ray S. (2019). Habit and addiction in the use of social networking sites: Their nature, antecedents, and consequences. Comput. Hum. Behav..

[77] Sumter S.R., Cingel D.P., Antonis D. (2018). “To be able to change, you have to take risks #fitspo”: Exploring correlates of fitspirational social media use among young women. Telemat. Inform..

[78] Moessner M., Feldhege J., Wolf M., Bauer S. (2018). Analyzing big data in social media: Text and network analyses of an eating disorder forum. Int. J. Eat. Disord..

[79] Pantic I. (2014). Online social networking and mental health. Cyberpsychol. Behav. Soc. Netw..

[80] Abbasi I., Drouin M. (2019). Neuroticism and Facebook addiction: How social media can affect mood?. Am. J. Fam. Ther..

[81] Köse Ö.B., Doğan A. (2019). The Relationship between Social Media Addiction and Self-Esteem among Turkish University Students. Addicta Turk. J. Addict..

[82] Sladek M.R., Salk R.H., Engeln R. (2018). Negative body talk measures for Asian, Latina(o), and White women and men: Measurement equivalence and associations with ethnic-racial identity. Body Image.

[83] Ceballos N., Czyzewska M. (2010). Body Image in Hispanic/Latino vs. European American Adolescents: Implications for treatment and prevention of obesity in underserved populations. J. Health Care Poor Underserved.

[84] Gordon K.H., Castro Y., Sitnikov L., Holm-Denoma J.M. (2010). Cultural body shape ideals and eating disorder symptoms among White, Latina, and Black college women. Cult. Divers. Ethn. Minority Psychol..

[85] Stokes D.M. (2016). Brown beauty: Body image, Latinas, and the media. Body Image.

